# Efficacy and Safety of a 6‐Month Placement of a Fully Covered Self‐Expanding Metal Stent for Refractory or Recurrent Hepaticojejunostomy Anastomotic Stricture via Balloon Enteroscopy‐Assisted Endoscopic Retrograde Cholangiopancreatography

**DOI:** 10.1002/deo2.70172

**Published:** 2025-07-01

**Authors:** Taro Hanaoka, Kosuke Okuwaki, Masafumi Watanabe, Tomohisa Iwai, Kai Adachi, Akihiro Tamaki, Junro Ishizaki, Yusaku Manabe, Masayoshi Tadehara, Rikiya Hasegawa, Takaaki Matsumoto, Hiroshi Imaizumi, Mitsuhiro Kida, Chika Kusano

**Affiliations:** ^1^ Department of Gastroenterology Kitasato University School of Medicine Kanagawa Japan

**Keywords:** balloon enteroscopy, biliary stricture management, endoscopic retrograde cholangiopancreatography, hepaticojejunostomy anastomotic stricture, self‐expanding metallic stent

## Abstract

**Objectives:**

Balloon enteroscopy‐assisted endoscopic retrograde cholangiopancreatography (BE‐assisted ERCP) is performed for hepaticojejunostomy anastomotic stricture (HJAS) after biliary‐enteric anastomosis. Although endoscopic balloon dilation (EBD) and plastic stent (PS) placement are commonly performed, they result in high recurrence rates. Studies have demonstrated the efficacy of fully covered self‐expanding metal stents (FCSEMS) in HJAS management; however, none have systematically evaluated the outcomes of 6‐month placement. Therefore, this retrospective study aimed to evaluate the efficacy and safety of a 6‐month FCSEMS placement for benign refractory or recurrent HJAS.

**Methods:**

We evaluated patients who underwent initial treatment with EBD alone or EBD plus PS placement via BE‐assisted ERCP between April 2015 and March 2024. Among them, patients with refractory or recurrent HJAS received 6‐month FCSEMS placements. The study outcomes were HJAS resolution, adverse events (AEs), and recurrence rates.

**Results:**

Among 92 patients who underwent initial EBD alone or EBD with PS placement, the HJAS resolution rate was 90.2%. The median follow‐up period after the initial treatment was 16.1 months, with recurrence observed in 48.2% of patients. Among the 34 patients with refractory or recurrent HJAS, FCSEMS placement achieved a resolution rate of 97.1% in a median of 182 days. AEs occurred in 14.7% of patients (moderate, *n* = 3; mild, *n* = 2) following FCSEMS placement. During a median follow‐up of 30.9 months, no recurrence was observed after HJAS resolution using FCSEMS.

**Conclusions:**

The 6‐month FCSEMS placement for refractory or recurrent HJAS showed high efficacy and safety, indicating its potential as a preferred treatment option.

## 1 Introduction

Hepaticojejunostomy anastomotic stricture (HJAS) is a well‐recognized late complication of biliary‐enteric anastomosis, occurring in 8%–13% of patients [1, 2, 3]. Historically, percutaneous transhepatic biliary drainage (PTBD) or surgical revision has been the primary treatment of HJAS in patients with surgically altered anatomy (SAA) due to the technical difficulty in reaching the anastomotic site. The advent of balloon enteroscopy (BE) [4] has enabled BE‐assisted endoscopic retrograde cholangiopancreatography (BE‐assisted ERCP) to become a viable therapeutic approach for HJAS [5, 6, 7, 8, 9, 10, 11].

Standard BE‐assisted ERCP procedures for HJAS include endoscopic balloon dilation (EBD) alone or in combination with plastic stent (PS) placement. However, these approaches are associated with high recurrence rates (8.3%–53%) [5, 6, 7, 8, 10, 11]. Recently, fully covered self‐expanding metal stents (FCSEMS) with a larger lumen diameter than that of PS have shown superior efficacy in resolving HJAS compared with conventional methods [12, 13, 14, 15, 16, 17]. Most studies have reported a median placement duration of 2–3 months [12, 14, 15, 17]. Studies on benign extrahepatic biliary strictures suggest that a 6‐month FCSEMS placement yields better outcomes than a 3‐month placement [18, 19]. Therefore, similar to benign extrahepatic biliary strictures, a 6‐month placement might offer improved outcomes of HJAS. However, the optimal stent indwelling duration, patient selection criteria, and long‐term outcomes of FCSEMS in patients with HJAS have not been adequately studied.

This retrospective study aimed to investigate the efficacy and safety of a 6‐month FCSEMS placement for refractory or recurrent HJAS managed with BE‐assisted ERCP.

## 2 Methods

### 2.1 Study Design

This single‐center retrospective study complied with the principles of the Declaration of Helsinki (1975, revised in 2000) and was approved by the Institutional Review Board (approval number: B24‐026). Owing to the retrospective nature of this study, informed consent was waived, and patient information was disclosed using an opt‐out approach.

### 2.2 Patient Eligibility

Patients who underwent initial BE‐assisted ERCP for symptomatic benign HJAS at Kitasato University Hospital between April 2015 and March 2024 were enrolled in this study. Patients in whom the hepaticojejunostomy site could not be reached or identified or those with complete anastomotic obstruction, were excluded from the study.

### 2.3 BE‐assisted ERCP Procedures

BE‐assisted ERCP was performed under conscious sedation using intravenous pethidine hydrochloride and midazolam. A short‐type single‐balloon enteroscope (SIF‐H290S; Olympus Medical Systems, Tokyo, Japan) was used. A SIF‐Q260 endoscope (Olympus Medical Systems) was used in patients for whom reaching the hepaticojejunostomy site was difficult.

**FIGURE 1:**
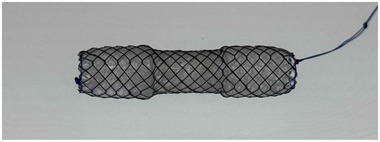
Fully covered self‐expanding metal stent: A representative dumbbell‐shaped FCSEMS used in this study, designed to prevent migration. All stents had a diameter of 10 mm at both ends and 8 mm at the central portion. FCSEMS, fully covered self‐expanding metal stent.

**FIGURE 2:**
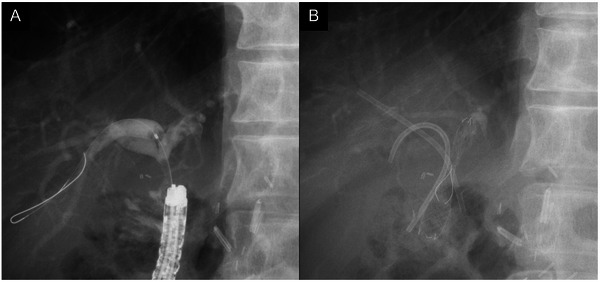
FCSEMS placement with PS for HJAS: (A) Cholangiographic image in BE‐assisted ERCP of a case of a short distance between the HJAS and confluence of the hepatic ducts. (B) After placing a 7‐Fr PS in both the anterior and posterior segments to prevent segmental cholangitis in an undrained area, an FCSEMS (10‐mm diameter × 30‐mm length) was deployed in the left hepatic duct. FCSEMS, fully covered self‐expanding metal stent; PS, plastic stent; HJAS, hepaticojejunostomy anastomotic stricture; BE‐assisted ERCP, balloon enteroscopy‐assisted endoscopic retrograde cholangiopancreatography.

**FIGURE 3:**
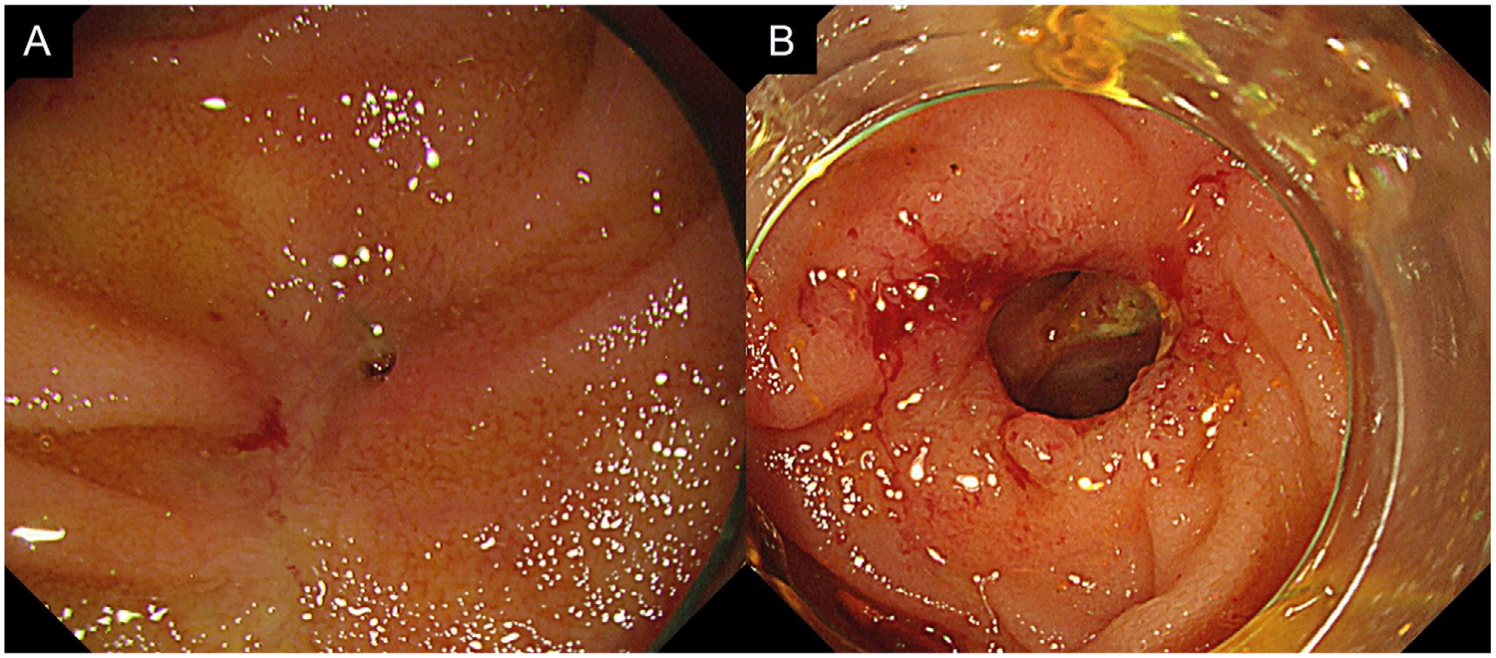
Endoscopic images before and after FCSEMS placement for HJAS: A representative case showing endoscopic images before (A) and after (B) FCSEMS placement, demonstrating resolution of HJAS achieved by endoscopic intervention. FCSEMS, fully covered self‐expanding metal stent; HJAS, hepaticojejunostomy anastomotic stricture.

Biliary cannulation was achieved using either a wire‐loaded or wire‐guided technique. After successful cannulation, cholangiography was performed with 30% amidotrizoate acid (Urografin, Bayer Yakuhin Ltd., Osaka, Japan). Before FCSEMS placement, balloon dilation of the HJAS was performed with a balloon catheter (REN, Kaneka Medix Corp., Osaka, Japan) when necessary. The FCSEMS used in all cases was the BONASTENT M‐intraductal (Sci‐Tech Inc., Seoul, South Korea) (Figure 1). To prevent segmental cholangitis, one or two 7‐Fr PS were placed as needed before FCSEMS placement (Figure 2A,B). FCSEMS removal was scheduled 6 months after placement via BE‐assisted ERCP (Figure 3). After FCSEMS placement, the patients underwent scheduled follow‐up, including periodic abdominal radiography for detection of spontaneous distal migration. In cases of migration with a concomitant PS, SBE‐ERCP was performed for stent retrieval and assessing HJAS resolution.

### 2.4 Definitions

Symptomatic HJAS was defined as intrahepatic bile duct dilation with biliary stasis detected on imaging (abdominal ultrasound, computed tomography, or magnetic resonance imaging), along with abnormal liver function tests and/or jaundice. HJAS resolution was defined as the absence of recurrence within 30 days after stent removal, according to the TOKYO 2024 criteria [20]. When EBD alone was performed, resolution was defined as the absence of recurrence within 30 days after the procedure. HJAS recurrence was defined based on the same criteria as those used for symptomatic HJAS described above. In patients who underwent FCSEMS placement, follow‐up was scheduled on the date of stent removal; in cases of spontaneous migration, follow‐up was started on the date of PS removal; for patients receiving no PS, follow‐up was considered on the date of confirming FCSEMS migration. Follow‐up ended upon HJAS recurrence or, if absent, on December 31, 2024. Treatment outcomes such as technical success and adverse events (AEs) related to FCSEMS placement were evaluated based on the TOKYO 2024 criteria [20].

### 2.5 Statistical Analyses

The time to HJAS recurrence was analyzed using the Kaplan–Meier method. Statistical analyses were performed using Bell Curve for Excel, version 4.08 (Social Survey Research Information Co. Ltd.).

## 3 Results

### 3.1 Patient Characteristics

**FIGURE 4:**
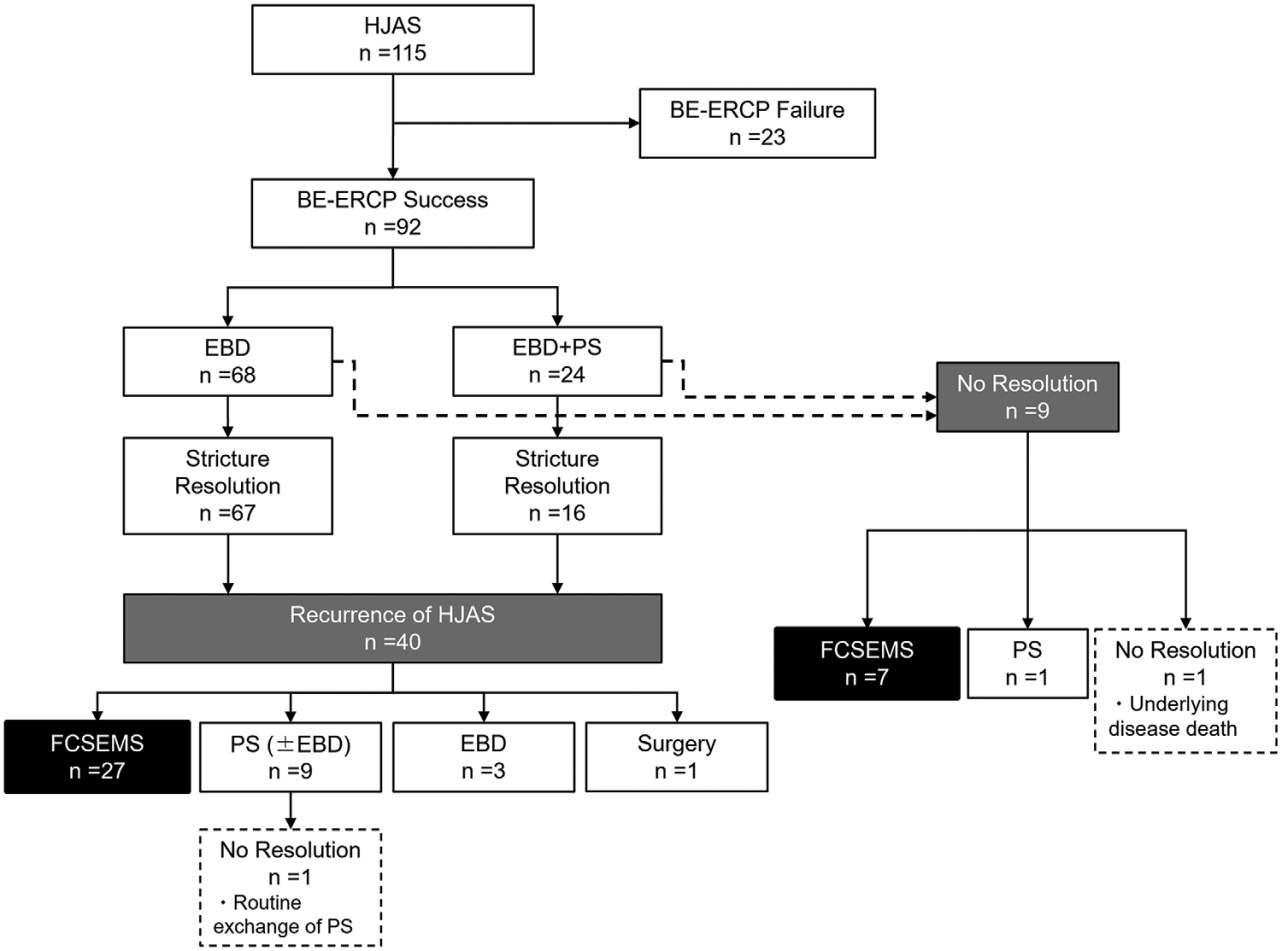
Flowchart of patient enrollment: BE‐ERCP, balloon enteroscopy‐assisted endoscopic retrograde cholangiopancreatography; EBD, endoscopic balloon dilatation; PS, plastic stent, FCSEMS, fully covered self‐expanding metal stent.

Between April 2015 and March 2024, 115 patients at Kitasato University Hospital underwent initial BE‐assisted ERCP for symptomatic benign HJAS. After excluding 23 patients (eight in whom the endoscope could not reach the hepaticojejunostomy site, 8 in whom the anastomotic site could not be identified, and seven with complete anastomotic obstruction), data from a total of 92 patients were included in the analysis (Figure 4).

The treatment strategies for HJAS included EBD alone (68 patients, 73.9%) and combined EBD with PS placement (24 patients, 26.1%). Among these, 83 patients (90.2%) achieved HJAS resolution following the initial treatment. Among the nine patients in whom HJAS resolution was not achieved, seven underwent FCSEMS placement, one underwent PS replacement with successful resolution at 6 months, and one continued PS placement but succumbed to recurrent pancreatic cancer 5 months later.

Among the 83 patients who initially achieved HJAS resolution, the median follow‐up period was 16.1 months (interquartile range [IQR]: 5.4–29.4 months), with recurrence observed in 40 patients (48.2%). The median time to HJAS recurrence was 6.8 months (IQR: 3.7–20.6 months). The treatments of recurrent HJAS in these 40 patients included FCSEMS placement (27 patients, 67.5%), scheduled PS exchange (nine patients, 22.5%), EBD alone (three patients, 7.5%), and surgical intervention (one patient, 2.5%).

Finally, 34 patients underwent FCSEMS placement, including seven patients with persistent HJAS after initial treatment and 27 with recurrent HJAS following initial resolution.

### 3.2 Clinical Outcomes of FCSEMS Placement for HJAS

Table 1 summarizes the baseline characteristics of the 34 patients who underwent FCSEMS placement. The outcomes of FCSEMS placement are summarized in Table 2. The technical success rate of FCSEMS placement was 100%. Among the 34 patients, 32 (94.1%) underwent concomitant PS placement. All PSs were 7‐Fr in diameter, with one and two PSs used in 28 and four patients, respectively. The median indwelling duration of the FCSEMS was 182 days (IQR: 137.3–186.8 days). AEs were observed in five patients (14.7%), all classified as late complications, including recurrent biliary obstruction (RBO) in three patients (8.8%) and non‐RBO complications in two patients (5.9%). The RBO cases comprised stent occlusion (moderate) in two patients (occurring at 123 and 133 days post‐placement) and proximal stent migration (moderate) in one patient (165 days post‐placement). Non‐RBO AEs included mild non‐occlusion cholangitis in two patients. During the FCSEMS indwelling period, spontaneous stent migration (distal migration) occurred asymptomatically in 12 patients (35.3%). Among these, eight cases (66.7%) occurred more than 3 months after FCSEMS placement. The median time to spontaneous migration was 133 days (IQR: 84.5–174 days). All FCSEMS were successfully endoscopically removed from the remaining 22 patients. HJAS resolution was achieved in 33 patients (97.1%) after FCSEMS placement. One patient in whom HJAS resolution was not achieved underwent an additional placement of a 7‐Fr PS for 123 days, resulting in eventual resolution. In addition, hyperplasia at the anastomotic site was observed in six patients (17.6%) at the time of FCSEMS removal or spontaneous stent migration.

**TABLE 1:** Characteristics of patients who underwent biliary drainage by fully covered self‐expanding metal stent (FCSEMS) for hepaticojejunostomy anastomotic stricture (HJAS).

*n* = 34	No. of patients (%)
Sex	
Male	22 (64.7)
Female	12 (35.3)
Median age, years [IQR]	70 [67–74]
Reconstruction method	
Modified Child's method after pancreaticoduodenectomy	27 (79.5)
Imanaga reconstruction after pancreaticoduodenectomy	1 (2.9)
Total pancreatectomy with Billroth II reconstruction	1 (2.9)
Extrahepatic bile duct resection with Roux‐en‐Y reconstruction	5 (14.7)
Reasons for surgery	
Benign diseases	9 (26.5)
Malignant diseases	25 (73.5)
Median time from surgery to the onset of the first anastomotic stricture, days [IQR]	347 [190.5–664.3]
Pretreatment leading up to FCSEMS implantation	
EBD	13 (38.2)
EBD + PS	21 (61.8)

Abbreviations: EBD, endoscopic balloon dilation; FCSEMS, fully covered self‐expanding metal stent; IQR, interquartile range; PS, plastic stent.

**TABLE 2:** Clinical outcomes of fully covered self‐expanding metal stent (FCSEMS) placement for hepaticojejunostomy anastomotic stricture (HJAS).

*n* = 34	No. of patients (%)
Technical success	34 (100)
Time from surgery to the onset of the first stricture	
< 6 months	8 (23.5)
≥ 6 months	26 (76.5)
FCSEMS length (mm)	
30	20 (58.8)
40	13 (38.2)
50	1 (3.0)
Number of PSs used in combination	
0	2 (5.9)
1	28 (82.4)
2	4 (11.7)
Adverse event	
Stent occlusion (moderate)	2 (5.9)
Proximal stent migration (moderate)	1 (3.0)
Non‐occlusion cholangitis (mild)	2 (5.9)
Distal stent migration before FCSEMS removal	
Yes	12 (35.3)
No	22 (64.7)
Successful FCSEMS removal^*^	
Yes	22 (100)
No	0
HJAS resolution	
Yes	33(97.1)
No	1 (2.9)

Abbreviations: FCSEMS, fully covered self‐expanding metal stent; HJAS, hepaticojejunostomy anastomotic stricture; PS, plastic stent.

^a^ Twelve patients were excluded due to distal migration of the FCSEMS.

**FIGURE 5:**
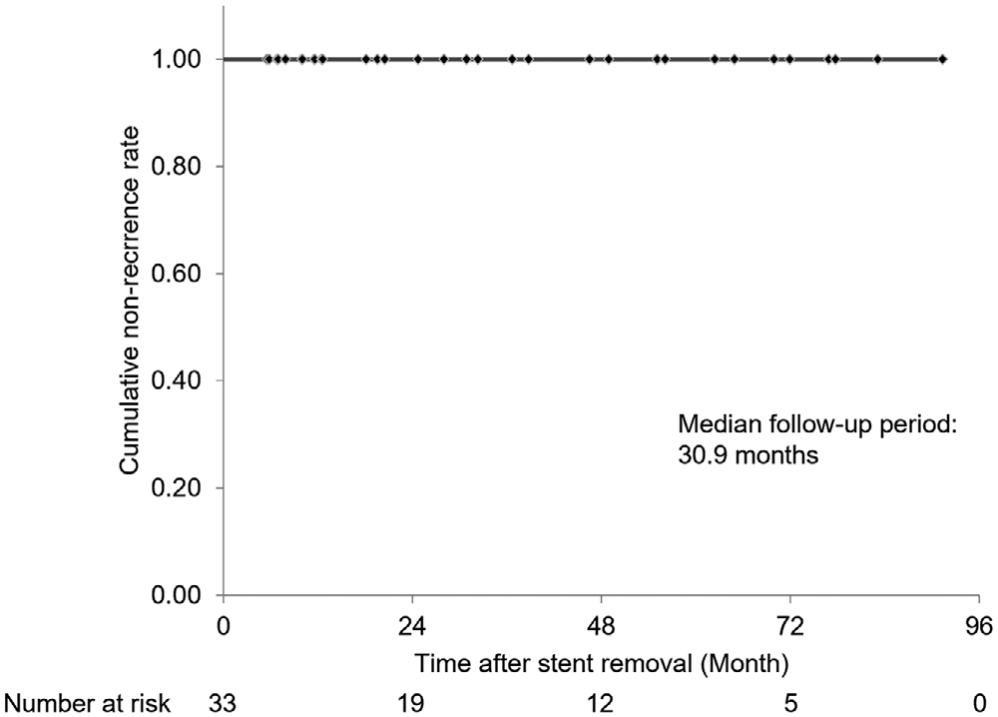
Kaplan–Meier curve of the non‐recurrence rate after stent removal: Kaplan–Meier curve showing the cumulative non‐recurrence rate after stricture resolution using FCSEMS for refractory benign HJAS. FCSEMS, fully covered self‐expanding metal stent; HJAS, hepaticojejunostomy anastomotic stricture.

During the median follow‐up period of 30.9 months (IQR: 11.6–56.1 months) after HJAS resolution, mild non‐occlusion cholangitis occurred in eight patients (24.2%), while intrahepatic stones requiring endoscopic treatment developed in two patients (6.1%). Of the 8 cases of mild non‐occlusive cholangitis, only one had exhibited hyperplasia at the time of HJAS resolution. Notably, no HJAS recurrence was observed during the follow‐up period (Figure 5).

## 4 Discussion

In addition to safety, achieving permanent stricture resolution is essential for the treatment of benign HJAS in patients with long life expectancy. The results of the present study demonstrated that FCSEMS placement for 6 months in patients with refractory or recurrent HJAS resulted in a high‐resolution rate of 97.1%, with only mild or moderate AEs. In addition, no HJAS recurrence was observed during a median follow‐up period of 30.9 months.

The advent of BE‐assisted ERCP has enabled the treatment of HJAS using EBD and PS placement. These treatments achieve initial HJAS resolution rates of 76.9%–100%; however, recurrence occurs in 8.3%–53% of patients for both EBD only and EBD combined with PS placement [5, 6, 7, 8, 10, 11]. To address this issue, recent studies have explored FCSEMS placement, with superior resolution rates [12, 13, 14, 15, 16, 17]. The reported median FCSEMS placement durations were 55 (6–99 days) [12], 94 (91–98 days) [14], 48 (16–136 days) [15], 51 (32–344 days) [16], and 98 (81–175 days) days [17]. Notably, studies on benign extrahepatic biliary strictures have indicated a direct correlation between stricture resolution and the duration of stent placement. Kahaleh et al. [18] reported that placement durations exceeding 3 months achieved significantly better resolution rates than did shorter placements, attributing this to sufficient time for remodeling of fibrosis or scar tissue. Saxena et al. [19] also observed that a 6‐month placement duration was superior to a 3‐month duration in terms of stricture resolution rate. We suggest that, similar to benign extrahepatic strictures, extended stent placement in patients with HJAS may enhance tissue remodeling at the anastomotic site, thereby contributing to improved long‐term outcomes and reduced recurrence. Based on these previous reports and considering that our study population comprised patients with refractory or recurrent HJAS, we opted for a 6‐month placement duration. This strategy might be particularly effective in patients at high risk of recurrence. A recent study identified early onset of HJAS after surgery as a significant risk factor for recurrence following balloon dilation, highlighting the need for more durable interventions in such cases [21].

The primary concerns about prolonged FCSEMS placement include sludge or stone formation, hyperplasia, and spontaneous distal migration. Sludge or stone formation was observed in 38.2% of patients, all of whom were effectively managed during scheduled stent removal. Hyperplasia was observed in 17.6% of patients. Although none of these patients experienced recurrence, hyperplasia might still represent a potential drawback of prolonged FCSEMS placement, particularly over longer‐term follow‐up. Spontaneous distal migration occurred in 35.3% of patients. Notably, 66.7% of these cases occurred more than 3 months after placement, suggesting a possible association with longer indwelling duration. Previous studies with shorter placement durations reported migration rates ranging between 6.3% and 30.8% [12, 14, 15, 16]. All migration cases in our study were asymptomatic; the stents had already been naturally expelled at the time of detection. Shibuya et al. [12] suggested that such migration may reflect improved stent patency and reduced need for reintervention. Although all distal migrations were asymptomatic and managed conservatively, the relatively high migration rate (35.3%)—with two‐thirds occurring beyond 3 months—raises concerns regarding the safety of prolonged FCSEMS placement in broader clinical application. Clinicians should remain vigilant, and future studies with larger cohorts are warranted to better elucidate the clinical implications of such migration. We scheduled follow‐up with abdominal radiography to allow early detection of migration and used SBE‐ERCP when necessary to retrieve remaining PSs and assess anastomotic status. These measures likely contributed to the absence of migration‐related AEs in our cohort. Nevertheless, clinicians should remain vigilant when using prolonged stent placement, as delayed migration may occur despite close monitoring. Regular follow‐up and timely intervention are essential for mitigating the risk of serious complications. The importance of such vigilance is supported by previous reports describing severe outcomes associated with migrated biliary stents [22, 23, 24].

Patients with refractory or recurrent HJAS in the present study underwent a 6‐month FCSEMS placement. The resolution rate and incidence of AEs were similar to those in previous reports in which the FCSEMS was placed for 2–3 months [12, 14, 15, 16, 17]. However, regarding long‐term outcomes after HJAS resolution with FCSEMS placement, our study had the longest median follow‐up period of 30.9 months compared with that in previous studies [12, 14, 15, 17], during which no recurrence was observed. In contrast, previous studies with FCSEMS placement periods of 2–3 months reported recurrence rates of 0%–27% [12, 14, 15, 16, 17]. One key implication of the present study is that the 6‐month FCSEMS placement may provide superior long‐term outcomes. Further follow‐up studies and comparative evaluations are required to confirm this hypothesis.

In the present study, 90.2% of HJAS cases resolved with initial treatment using EBD, EBD with PS placement. During a median follow‐up of 16.1 months, 51.8% of patients remained recurrence‐free, suggesting that these cases may not necessarily require FCSEMS placement. Among studies on the risk factors for recurrence after initial endoscopic treatment of HJAS, Yamauchi et al. [7] observed a higher recurrence rate when HJAS developed within 6 months post‐surgery, whereas Iwasa et al. [11] reported a higher recurrence rate when HJAS developed within 13.2 months post‐surgery. Based on the findings of this study, the favorable outcomes of 6‐month FCSEMS placement in cases of refractory or recurrent HJAS suggest that patients with initial treatment failure or recurrence, as well as those with a short interval between surgery and HJAS onset, may be appropriate candidates for a 6‐month FCSEMS placement. In addition, a recent study has demonstrated that preoperative self‐expanding metal stent placement might help maintain bile duct diameter ≥5 mm and reduce the risk of benign HJAS development after pancreaticoduodenectomy [25]. Such preventive strategies might be complementary to endoscopic management, particularly in high‐risk patients.

This study has several limitations. For instance, in the retrospective analysis, treatment decisions for refractory or recurrent HJAS were made at the discretion of the attending physician, leading to variability in therapeutic approaches, with a subset of patients receiving interventions other than FCSEMS placement. Additionally, among the patients who received FCSEMS placement, 10 underwent the procedure after only one prior endoscopic treatment. These patients may not have had truly refractory HJAS, which could have led to an overestimation of the resolution rate. However, considering the high efficacy of FCSEMS and the burden of repeated interventions, early use of FCSEMS after the first recurrence might still be a clinically reasonable strategy. Furthermore, as this was a single‐center study with a relatively small sample size, the ability to evaluate the efficacy and safety of FCSEMS placement in relation to patient characteristics and stent duration was limited. Consequently, larger multicenter comparative studies with more extensive cohorts are required to validate these findings. Nonetheless, despite these constraints, the results indicate that a 6‐month FCSEMS placement is a viable and promising therapeutic strategy for refractory HJAS, demonstrating both high efficacy and a favorable safety profile.

In conclusion, a 6‐month FCSEMS placement was effective in patients with refractory HJAS, achieving high resolution with no serious AEs observed, although the high rate of distal migration remains a concern. No recurrence was observed during the long‐term follow‐up. These findings suggest that prolonged FCSEMS placement may be a promising therapeutic strategy. However, further large‐scale studies are needed to compare stent placement duration and clarify long‐term outcomes.

## Ethics Statement

**Approval of the research protocol by an Institutional Review Board**: This single‐center retrospective study complied with the principles of the Declaration of Helsinki (1975, revised in 2000) and was approved by the Institutional Review Board of Kitasato University Hospital (Approval No. B24‐026).

## Consent

Because of the retrospective nature of this study, the requirement for written informed consent was waived; instead, patient information was disclosed to the public using an opt‐out approach.

## Conflicts of Interest

The authors declare no conflicts of interest.

## Clinical Trial Registration

Not applicable.

## Data Availability

Data supporting the findings of this study are available from the corresponding author upon request.
